# Leptin Level and Oxidative Stress Contribute to Obesity-Induced Low Testosterone in Murine Testicular Tissue

**DOI:** 10.1155/2014/190945

**Published:** 2014-04-14

**Authors:** Jian Zhao, Lingling Zhai, Zheng Liu, Shuang Wu, Liping Xu

**Affiliations:** ^1^Department of Pharmacology, Shenyang Pharmaceutical University, No. 103 Wenhua Road, Shenhe District, Shenyang, Liaoning 110016, China; ^2^Department of Child and Adolescent Health, School of Public Health, China Medical University, Shenyang, Liaoning 110001, China

## Abstract

*Objective*. This study evaluated the effects of obesity on the function of reproductive organs in male mice and the possible mechanism of male secondary hypogonadism (SH) in obesity. *Methods*. Ninety-six mice were randomly assigned to three groups: the control group, diet-induced obesity group, and diet-induced obesity resistant group for 8 weeks and 19 weeks. The effects of short- and long-term high-fat diet on the reproductive organs were determined by measuring sperm count and motility, relative testis weight, testosterone level, pathological changes and apoptosis of Leydig cells. Oxidative stress was evaluated by determining malondialdehyde, H_2_O_2_, NO levels, and GSH in testis tissues. CAT, SOD, GSH-Px and Nrf_2_ mRNA were measured by real-time PCR. *Results*. Short- and long-term high-fat diet decreased sperm count and motility, relative testis weight, testosterone level; decreased CAT, SOD, GSH-Px and Nrf_2_ mRNA expression; increased MDA, H_2_O_2_, NO and leptin levels; inhibited the activity of CAT and GSH-Px enzymes. Pathological injury and apoptosis of Leydig cells were found in testis tissue. *Conclusions*. Pathological damage of Leydig cells, oxidative stress in testis tissue, and high level of leptin may provide some evidence to clarify the mechanisms of male SH in obesity.

## 1. Introduction

There has been an increase in the prevalence of childhood obesity in both developed and developing countries [1]. In the United States, the incidence of childhood obesity is estimated to be 17%, or 12 million children aged between 2 and 19 years [1]. In addition, the prevalence of overweight and obesity is rising in China [2, 3]. Obesity is a multifactorial condition with syndromic and nonsyndromic variants [4–6] and has both immediate and long-term health consequences. Tracking of adiposity from childhood into adulthood is much stronger in obese children [5]. Unfortunately, there are few effective treatments for overweight and obese children [7–9].

It has been demonstrated that obesity has an impact on male reproduction [10]. Male obesity is associated with an increased incidence of low sperm concentration and a progressively low motile sperm count [11, 12]. Even in the absence of organic disease of the hypothalamo-pituitary unit, the prevalence of secondary (hypogonadotropic) hypogonadism (SH) in obese men has been demonstrated in several studies [10, 13, 14]. The pathogenesis and clinicopathological correlates of obesity-associated SH are incompletely understood on the basis of the current literature [15]. The mechanisms involved in the association between male SH and obesity are complex. However, male obesity per se is associated with lower plasma testosterone levels [14]. The development of reproductive organs and male secondary sexual characteristics is promoted by androgens. Spermatogenesis is closely related to androgen secretion. Reduced testosterone may contribute to male SH in obesity [16].

We hypothesize that the reasons for the decrease in testosterone are as follows. (1) Testosterone is mainly secreted in testicular Leydig cells and damage to testicular Leydig cells may contribute to decreased testosterone. (2) An increased number of adipose cells are found in obesity. Adipose cytokines such as leptin are secreted in adipose cells. The level of adipose cytokines is high and may suppress secretion of testosterone. (3) Oxidative stress is closely related to SH. Approximately 15% of couples attempting to conceive are clinically infertile and male factor infertility is involved in 50% of these cases [17]. Testicular oxidative stress appears to be a common feature in male infertility and is likely to play a significant role in male infertility [18]. However, there is no evidence of an association between oxidative stress and male SH in obese patients.

In this study, weanling mice were fed a high-fat diet for 8 weeks and 19 weeks. The effects of short- and long-term high-fat diet on the function and development of reproductive organs in male mice were determined, as our aim was to assess whether pubertal obesity influenced the function and development of reproductive organs in adults. In addition, the possible mechanism of male SH in obesity was evaluated.

## 2. Materials and Methods

### 2.1. Animals, Diet, and Experimental Procedures

96 4/5-week-old C57BL/6J male mice were obtained from the Experimental Animal Center, China Medical University, Shenyang, China. Mice were fed standard laboratory chow for the 1st week to allow them to adjust to their new environment. Then the mice were randomly assigned to a standard lab diet (10% of calories from fat, 20% of calories from protein, and 70% of calories from carbohydrates, 3.85 kcal/g) or a prepared high-fat diet, which contained 45% kcal from fat (the high-fat diet group) [19], for 8 or 19 weeks. The high-fat diet was made up of 73% standard chow diet plus 20% lard, 7% casein (Ao-boxing Biotech Company Ltd., Beijing, China), and trace amounts of multiple vitamins.

The animals were housed in a temperature- and humidity-controlled room (25 ± 2°C and 55 ± 10%, resp.) on a 12-hour light/dark cycle with free access to food and water. All experimental procedures conformed to the Institutional Guidelines for the Care and Use of Laboratory Animals of China Medical University, Shenyang, China, and to the National Institutes of Health Guide for Care and Use of Laboratory Animals (publication number 85-23, revised in 1985). All efforts were made to minimize the number of animals used and their suffering.

**Figure 1:**
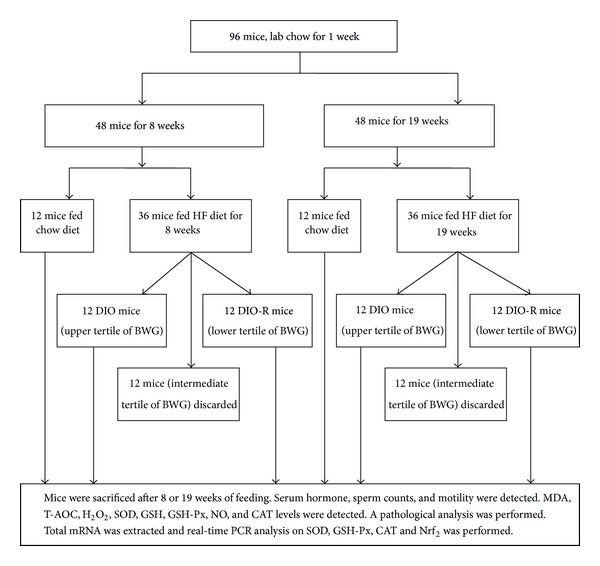
The flowchart of the animal experiment.

After 8 or 19 weeks on their respective diets, mice fed the high-fat diet were divided by body weight gain into DIO-R (diet-induced obesity resistant) and DIO (diet-induced obesity) mice, according to the method used by Levin and Keesey [20] shown in Figure 1. Twelve mice in the upper tertile of body weight gain (11.54 ± 0.42 g, 16.58 ± 1.39 g) were designated as DIO at 8 weeks and 19 weeks, respectively, and 12 mice in the lower tertile of body weight gain (BWG) (8.75 ± 1.24 g, 12.00 ± 1.23 g) as DIO-R at 8 weeks and 19 weeks, respectively. Mice with intermediate tertile body weight gain (*n* = 24) were excluded from this study. All mice in each group were sacrificed after 8 and 19 weeks of feeding.

### 2.2. Processing of Tissues and Assays

Body weight and food consumption were recorded. At 24 hours after receiving the last feed, animals were anesthetized with ether and blood samples were obtained from the vena cava. Serum was separated for measurement of the sex hormone, testosterone. Immediately after blood samples were collected, the epididymis was rapidly excised, and sperm count and motility were determined. Retroperitoneal fat, epididymal fat, epididymal tissue, and testis and seminal vesicles were obtained and weighed.

Six testicles from each group were prepared as a 5% or 10% homogenate in order to determine MDA, T-AOC, H_2_O_2_, SOD, GSH, GSH-Px, CAT, and NO levels. Six testicles from each group were immediately frozen at −80°C for gene expression studies.

Six testicles from each group were prepared for transmission electron microscopy. The testis was cut into fragments (1 mm × 1 mm × 1 mm), fixed in 2.5% glutaraldehyde with 0.1 M phosphate buffer (pH 7.2), postfixed in 1.0% osmium tetroxide (OsO_4_), dehydrated in a progressive ethanol and acetone solution, embedded in Epon 812, sectioned using an LKB ultramicrotome, and stained with uranyl acetate followed by lead citrate, then observed by H-600 microscopy, and photographed.

The remaining six testicles from each group were prepared for pathological analysis.

All the contents and enzyme activities were normalized to the protein which was measured by the method of Lowry [21], using bovine serum albumin (BSA) as standard. Each sample was tested in triplicate.

### 2.3. Cauda Epididymal Sperm Count and Motility Measurements

Male C57BL/6J mice were weighed and anesthetized at 8 and 19 weeks. The left epididymis was immediately removed. The epididymis and vas deferens were dissected away from the fat. In a 6-well plate, the epididymis and vas deferens from each animal were placed in a well containing 1.0 mL of M2 buffer. The epididymis was then cut at the junction between the corpus and cauda epididymis, and the cauda was placed into a well with 1.0 mL of M2 buffer. Several cuts were made in the cauda epididymis with scissors, and sperm was gently pressed. Sperm was also obtained from the vas deferens in a separate well and then removed from the plate. The pressed sperm from the cauda epididymis was then collected in an Eppendorf tube. Using a hemocytometer (15 mL per side), sperm counts were determined as the number of sperm per microliter.

Sperm count and motility were assessed in accordance with WHO guidelines [22] (⩾200 sperm counted for each sample). Sperm count was determined using a hemocytometer. Sperm motility was assessed blindly under a light microscope by classifying 200 sperms per animal as either progressive motile, nonprogressive motile, or immotile. Motility was then expressed as percent of total motile (progressive motile and nonprogressive motile sperm).

### 2.4. Pathological Analysis

Testicular tissue was removed and the tissue was fixed with 4% paraformaldehyde. Part of the testicular tissue from each mouse was cut into 4 *μ*m thick pieces. Hematoxylin and eosin (HE) staining was performed for morphological observation using an AX-70 microscope (Olympus, Japan).

The testicular tissue samples were dehydrated in a graded series of ethanol solution, embedded in paraffin, and coronal sectioned using a section cutter (Leica, USA) at a thickness of 4 *μ*m. The testicular sections were processed for apoptosis TUNEL staining and were prepared for subsequent microscopic mounting (each group, *n* = 6, 3 sections per animal) as follows. (1) Paraffin sections were dewaxed. (2) 1% triton-100 was added for 15 min, and all slides were rinsed with PBS 3 times. (3) The enzyme was inactivated with 3% H_2_O_2_-methanol for 15 min; all slides were rinsed with PBS 3 times. (4) 100 *μ*L TUNEL reaction mixture (or 100 *μ*L control solution for negative control) was added to each slide and the slides were incubated in a humid chamber for 60 min at 37°C and then washed with PBS 3 times. (5) The slides were wiped around the tissue and 100 *μ*L streptavidin-HRP was added to each sample. The slides were incubated in a humid chamber for 30 min at 37°C. The slides were washed once with PBS and then washed 3 times with 100 mM tris buffer and pH 8.2 for 5 min at room temperature. (6) DAB chromogenic was added. (7) dH_2_O was added to stop the color reaction. Apoptotic cells were stained brown and normal cells were blue-violet. The apoptotic rate = (apoptotic cells/total number of cell) × 100%. A pathological analysis was performed in testis tissue. Total mRNA was extracted from testis tissue and real-time PCR analysis on SOD, GSH-Px, CAT and Nrf_2_ was performed.

### 2.5. MDA Assay

The testis homogenate was added to the reaction mixture containing 0.1 mol/L phosphate buffer and 0.1 mol/L FeCl_3_ in a total volume of 1.0 mL (pH = 7.4). The reaction was stopped by the addition of 1.0 mL 10% trichloroacetic acid (TCA), followed by 1.0 mL 0.67% TBA, and the tubes were placed in a boiling water bath for 20 min. The tubes were then moved to an ice bath and the contents were centrifuged at 2500 g for 10 min. The amount of MDA formed in each of the samples was assessed by measuring the optical density of the supernatant at 535 nm using tetraethoxypropane (TEP) as a standard. MDA content was expressed as nmol*·*mg^−1^ protein.

### 2.6. Glutathione Assay

GSH in testicular homogenate was determined by the reaction with 5,5′-dithiobis-(2- Nitrobenzoic Acid) (DTNB). Briefly, 0.9 mL of 10% testicular homogenate was added to 0.1 mL of 50% TCA, and the samples were centrifuged at 3000 rpm for 15 min. Then 0.1 mL of supernatant was added in 4.4 mL of 0.1 M PBS and 0.5 mL of 0.04% DTNB to a total volume of 5.0 mL (pH 7.4). The absorbance of the solution was measured spectrophotometrically at 412 nm. The content of GSH was expressed as mg GSH g^−1^ protein.

### 2.7. GSH-Px, CAT, SOD, H_2_O_2,_ NO, and T-AOC Assays

Assay kits for GSH-Px, CAT, SOD, H_2_O_2,_ NO, and T-AOC were provided by Jiancheng Bioengineering Institute (Nanjing, China). The GSH-Px, CAT, SOD, H_2_O_2_, NO, and T-AOC contents were measured using these kits following the manufacturer's instructions. The GSH-Px, CAT, SOD, and T-AOC contents were expressed as U·mg^−1^ protein. H_2_O_2_ content was expressed as mmol g^−1^ protein. NO content was expressed as *μ*mol g^−1^ protein.

### 2.8. Isolation of RNA and Real-Time PCR Analysis

Total mRNA was extracted from mouse testicular tissue in each group. Real-time PCR was performed with a SYBR green PCR kit (TaKaRa Biotechnology Co., Ltd., Dalian, China) using a real-time PCR system (Applied Biosystems 7500 Real-Time PCR system). Total RNA was extracted from testis using Trizol (TaKaRa Biotechnology Co., Ltd. Dalian, China). The quantity and integrity were characterized using a UV spectrophotometer. OD260/OD280 of total RNA was between 1.6 and 1.8.

Semiquantitative reverse transcription was performed using the PrimeScript RT reagent kit with DNA eraser. Reverse transcription was performed using an Applied Biosystems 2720 Thermal Cycler (Applied Biosystems, Singapore). Remove DNA reaction of 1 *μ*g of total RNA was performed in a final volume of 10 *μ*L, using 2.0 *μ*L 5x gDNA Eraser Buffer, 1.0 *μ*L gDNA Eraser and RNase-Free dH_2_O. The reaction was incubated at 42°C for 2 min. Reverse transcription was performed in a final volume of 20 *μ*L, using 10 *μ*L outcome of remove DNA reaction, 1.0 *μ*L Primescript RT Enzyme Mix I, 4.0 *μ*L 5x Primescript buffer 2 and 1.0 *μ*L RT prime Mix and RNase-Free ddH_2_O. The reaction was incubated at 37°C for 15 min, then at 85°C for 5 s.

The gene-specific primers were designed by TaKaRa Co. All primers are listed in Table 1. The 50 *μ*L PCR reaction mixture contained 25 *μ*L of 2x PCR buffer, 2 *μ*L of PCR forward primer (10 *μ*M), 2 *μ*L of PCR reverse primer (10 *μ*M), 1 *μ*L of ROX Reference Dye II (50x), 4 *μ*L of template DNA, and 16 *μ*L dH_2_O. The initial denaturation was carried out at 95°C for 30 s, followed by amplification in 40 cycles, 95°C for 3 s, and 60°C for 34 s using the 7500 Real-Time PCR system (BD Co., USA). The relative expression analysis was carried out using the 2−ΔΔCT method. For which ΔCT (test) = CT (target, test) − CT (reference, test), ΔCT (calibrator) = CT (target, calibrator) − CT (reference, calibrator), and ΔΔCT = ΔCT (Test) − ΔCT (calibrator). The relative expression was calculated by 2−ΔΔCT. The raw data were normalized to those of the housekeeping gene, *β*-actin. All reactions were performed in triplicate. Following amplification, a melting curve analysis was performed to verify the correct product according to its specific melting temperature (Tm) [23].

### 2.9. Statistical Analysis

Significant differences between obtained values (mean ± SD) were determined by one-way analysis of variance (ANOVA) followed by the least significant difference (LSD) multiple comparison test. A *P* value of <0.05 was considered significant.

## 3. Results

### 3.1. Body Weight and Body Fat

A significant difference in body weight was observed in male C57BL/6J mice fed the high-fat diet in comparison to age-matched littermates fed a normal diet at 8 weeks and 19 weeks (*P* < 0.01).

**Figure 2:**
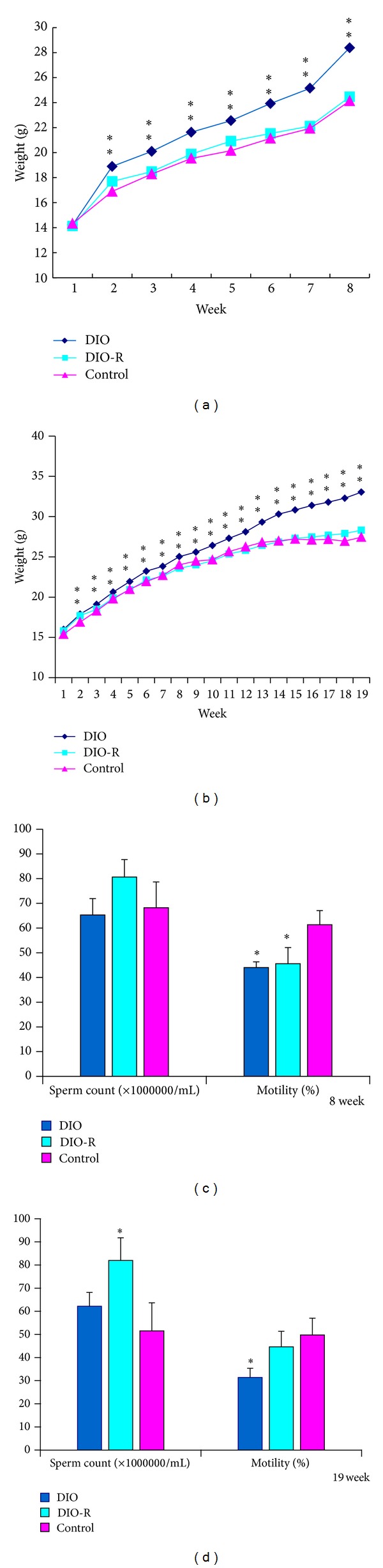
Effect of high-fat feeding on body weight and sperm count, motility in 8 weeks and 19 weeks. All data are expressed as means ± SD; *P < 0.05, **P < 0.01, versus control group. (a) Bodyweights at the end of 8 weeks of control mice (*n* = 12) fed a 10% fat diet and diet-induced obesity (DIO) mice (*n* = 12) and diet-induced obesity resistant (DIO-R) mice (*n* = 12) fed a 45% fat diet for the times indicated, (b) bodyweights at the end of 19 weeks of control mice (*n* = 12) fed a 10% fat diet and diet-induced obesity (DIO) mice (*n* = 12) and diet-induced obesity resistant (DIO-R) mice (*n* = 12) fed a 45% fat diet for the times indicated, (c) sperm count and motility in control (*n* = 6), DIO-R (*n* = 6), and DIO mice (*n* = 6) at the end of 8 weeks, and (d) sperm count and motility in control (*n* = 6), DIO-R (*n* = 6), and DIO mice (*n* = 6) at the end of 19 weeks.

Mice fed the high-fat diet showed increased body weight averaging 28 g at 8 weeks in the DIO group, whereas mice in the DIO-R group averaged 24 g, and littermates fed the normal diet averaged 24 g at the same age (Figure 2(a)). Mice fed the high-fat diet showed increased body weight averaging 33 g at 19 weeks in the DIO group, whereas mice in the DIO-R group averaged 28 g, and littermates fed the normal diet averaged 27 g at the same age (Figure 2(b)).

Absolute and relative retroperitoneal (Ret) and epididymal (Epi) fat pads were significantly higher in the DIO group and DIO-R group versus the control group mice at 8 weeks and 19 weeks (Table 2) (*P* < 0.01).

### 3.2. Effect of Diet on Reproductive Organs

As shown in Table 2, DIO, DIO-R, and control mice did not exhibit significant differences in the absolute average weight of testes or epididymis at 8 weeks and 19 weeks. However, there was a significant decrease in the relative testis weight in the DIO group compared to the control group at 8 weeks (*P* < 0.05). There was a significant decrease in the relative testis weight and epididymal weight in the DIO group compared with the control group at 19 weeks (*P* < 0.05). The relative testis weight in the DIO group was lower than that in the DIO-R group at 8 weeks and 19 weeks. However, the absolute weight of seminal vesicles in the DIO and DIO-R groups was significantly higher than the normal group at 8 weeks (*P* < 0.05). The relative weight of seminal vesicles in the DIO and normal groups was lower than that in the DIO-R group at 8 weeks (*P* < 0.01).

### 3.3. Effect of Diet on Sperm Count and Motility

DIO, DIO-R, and control male mice exhibited no disparities in morphology or total sperm count collected from the cauda epididymis at 8 weeks. However, the DIO and DIO-R group exhibited a notable 28% decrease in sperm motility at 8 weeks (*P* < 0.05) (Figure 2(c)). At 19 weeks, there was a significant increase in total sperm count in the DIO-R group compared with the control group (*P* < 0.05). In addition, the DIO group exhibited a notable 37% decrease in sperm motility (*P* < 0.05) (Figure 2(d)).

### 3.4. Effect of Diet on Serum Testosterone and Leptin

As shown in Table 3, DIO and DIO-R mice exhibited decreased fasting levels of testosterone at 8 weeks. Significantly higher serum testosterone levels were observed in the control group when compared to the DIO and DIO-R group at 8 weeks and 19 weeks (*P* < 0.05). And significantly lower serum leptin levels were observed in the control group when compared to the DIO and DIO-R group at 8 weeks and 19 weeks (*P* < 0.05, *P* < 0.01).

### 3.5. Effect of Diet on Pathological Changes

**Figure 3:**
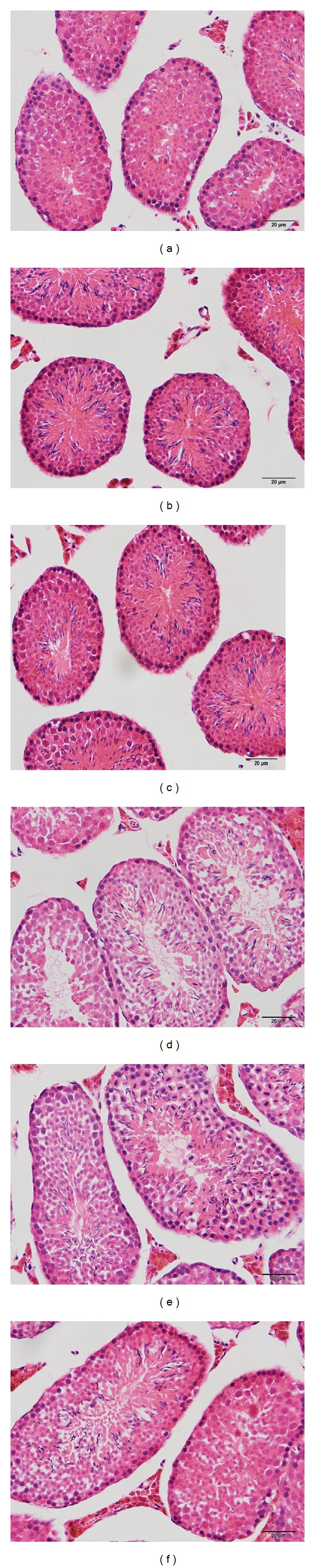
Light microphotographs showed the morphological changes of testicular cells after 8 and 19 weeks of high-fat diet. Photographs of control at 8 weeks (a), DIO group at 8 weeks (b), DIO-R group at 8 weeks (c), control group at 19 weeks (d), DIO group at 19 weeks (e), and DIO-R group (f) at 19 weeks were in this figure. The sections were stained with HE staining. Magnification ×40.

**Figure 4:**
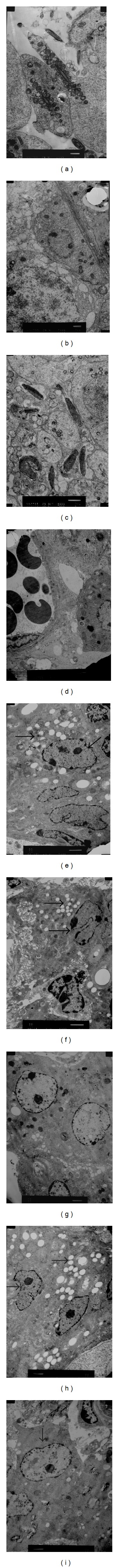
Electron microscopy of mice testes following the diet-induced fat for 8 weeks and 19 weeks shows a large number of lipid droplets, irregular karyotype, and heterochromatin side set (arrow) of Leydig cells ((e), (f), (h), and (i)) compared to control ((d) and (g)). (a) Normal sperm formation in the control group, (b) normal morphology of basement membrane, spermatogonia, and primary spermatocytes in the DIO group, (c) normal morphology of spermatids, (d) normal stromal cells and blood vessels, (e) in Leydig cells: a large number of lipid droplets, irregular karyotype, and heterochromatin side set found in DIO group at 8 weeks, (f) in Leydig cells: a large number of lipid droplets, irregular karyotype, and heterochromatin side set found in DIO-R group at 8 weeks, (g) normal Leydig cells, a small amount of fat vacuolar found in control group at 19 weeks, (h) in Leydig cells: a large number of lipid droplets, irregular karyotype, and heterochromatin side set found in DIO group at 19 weeks, and (i) in Leydig cells: irregular karyotype and heterochromatin side set found in DIO-R group at 19 weeks.

To confirm the effects of exposure to the high-fat diet on morphological changes in testicular tissue, HE staining and electron microscopy were performed. Light microphotographs showed morphological changes in testicular cells after 8 and 19 weeks of the high-fat diet (Figure 3). No significant difference was observed between the groups. Electron microscopy of mouse testes was performed following the high-fat diet for 8 weeks and 19 weeks. Sperm formation was normal in the control group, and normal morphology of spermatids and normal stromal cells and blood vessels were found in all groups. However, a large number of lipid droplets, irregular karyotype, and heterochromatin side set were found in the DIO and DIO-R groups at 8 weeks and 19 weeks (Figure 4).

**Figure 5:**
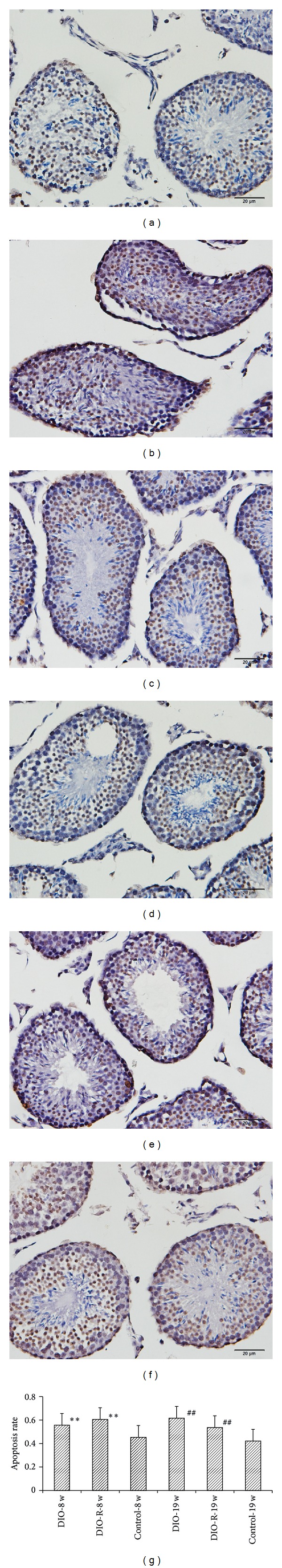
The figure showed the TUNEL apoptosis assay in Leydig cells after 8-week and 19-week high-fat diet. Photographs of control at 8 weeks (a), DIO group at 8 weeks (b), DIO-R group at 8 weeks (c), control group at 19 weeks (d), DIO group at 19 weeks (e), and DIO-R group (f) at 19 weeks were in this figure. The sections were stained with DAB, and the magnification was set at ×40. Data are mean ± SD for six animals in each group. The effects of 8-week and 19-week high-fat diet on Leydig cells apoptotic rate (g) were also shown in this figure. **P < 0.01 denotes statistical significance compared with control-8w group; ^##^ *P* < 0.01 denotes statistical significance compared with control-19w group.

To determine testicular tissue apoptosis caused by high-fat diet, the TUNEL apoptosis assay was used to quantify the rate of cell apoptosis. This assay showed that few cells were viable (brown staining) in controls (Figures 5(a) and 5(d)). Many apoptotic cells (brown staining) were found in the DIO-8w, DIO-R-8w, DIO-19w, and DIO-R-19w groups (Figures 5(b), 5(c), 5(e), and 5(f)). The high-fat diet increased apoptosis rates by 1.24-, 1.36-, 1.48-, and 1.29-fold as compared to the normal control, respectively (Figure 5).

### 3.6. Effect of Diet on Oxidative Stress in Testis Tissue

The effect of diet on oxidative stress biomarkers is shown in Table 4. At 8 weeks, diet increased the levels of MDA in testis tissue to 81% and 14% of control in the DIO group and DIO-R group, respectively. When compared with the control group, H_2_O_2_ levels were increased to 1.53-fold and 1.55-fold in the DIO group and DIO-R group, respectively. NO levels were increased to 1.68-fold in the DIO group and DIO-R group, respectively. A significant decrease in the activity of CAT was observed in the DIO group and DIO-R group compared to the control group (*P* < 0.05) (Table 4). However, there was no difference in T-AOC, GSH, SOD, and GSH-Px levels in testis tissues compared to the control group.

At 19 weeks, it was found that diet increased the levels of MDA in testis tissue to 89% and 138% of the control in the DIO and DIO-R group, respectively. When compared with the control group, H_2_O_2_ levels were increased to 1.32-fold and 1.23-fold in the DIO group and DIO-R group, respectively. The NO levels in the DIO and DIO-R groups were significantly higher than those in the control group (*P* < 0.05). Although T-AOC levels decreased in both DIO and DIO-R groups, only T-AOC level in the DIO group was significantly lower than that in the control group (*P* < 0.05). The GSH level in the DIO group was significantly higher than that in the control group (*P* < 0.05). A significant decrease in the activity of CAT and GSH-Px was found in the DIO and DIO-R groups compared to the control group (*P* < 0.05) (Table 4). However, there was no difference in SOD levels in testis tissues compared with the control group.

### 3.7. The Effect of Diet on Antioxidant Gene Expression

**Figure 6:**
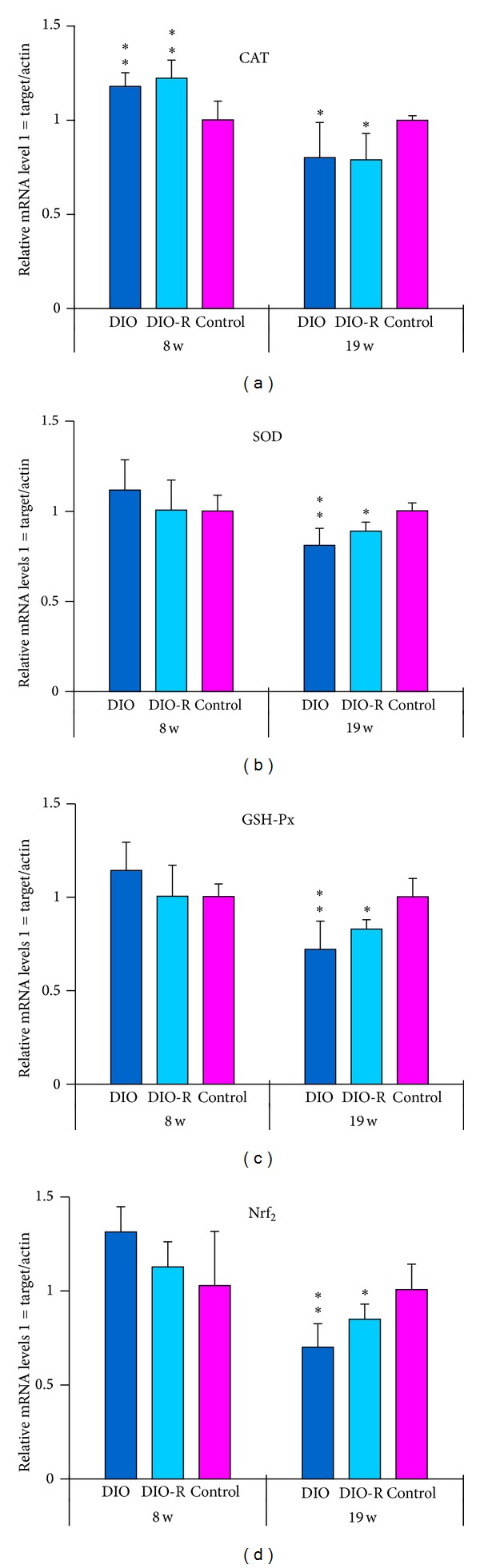
The alteration of CAT mRNA (a), SOD mRNA (b), GSH-Px mRNA (c), and Nrf_2_ mRNA (d) in testicular tissue after high-fat diet at 8 and 19 weeks was shown in this figure. Data are mean ± SD for 6 animals in every group and each RNA preparation was run three times by real-time PCR; *P < 0.05 and **P < 0.01 denote statistical significance compared with control group.

The effect of diet on the gene expression level of SOD, GSH-Px, catalase, and Nrf_2_ in testis tissue was confirmed by real-time PCR.  Figure 6 shows the effect of diet on mRNA expression of the antioxidant genes SOD, GSH-Px, catalase, and Nrf_2_ in testis tissue by quantitative detection of gene expression. At 8 weeks, the DIO and DIO-R groups showed significant upregulation of the catalase gene. Other gene expressions were similar between the 3 groups. However, at 19 weeks, the DIO group showed significant downregulation of all the studied genes.

## 4. Discussion

Testosterone is the most important sex hormone in males and plays a critical role in testis development, spermatogenesis, and maintenance of normal masculinization.

During puberty, testosterone is involved in many of the processes in the transition from a boy to manhood, including healthy development of male sex organs. Throughout adulthood, this hormone also plays an important role in maintaining libido and sperm production.

Disorders of the testes are caused by too little testosterone production. Obesity causes hormonal modification and hypogonadism [24]. Saboor Aftab et al. found that male obesity per se is associated with a lower plasma testosterone level [15]. In the present study, when mice were fed the high-fat diet for 8 or 19 weeks, we found the following: (1) lower plasma testosterone level; (2) decline in sperm motility; (3) Leydig cells damaged with increased apoptosis; (4) decreased testis and epididymis relative coefficient. Pubertal obesity may influence the function and development of reproductive organs in adults. Male obesity may cause hypogonadism which is a testicular disorder associated with low testosterone. Lower plasma testosterone level plays an important role in male hypogonadism caused by obesity.

In this study, we will discuss the mechanism of low testosterone levels caused by fat intake.

(*1) Pathologic Changes in Leydig Cells and Low Testosterone Level Induced by Fat*. Leydig cells, also known as interstitial cells of Leydig, produce testosterone in the presence of luteinizing hormone (LH). A large amount of smooth endoplasmic reticulum (SER) is present in Leydig cells, and there is an abundance of the cholesterol synthesis enzyme in the SER. The SER has a strong ability to synthesize cholesterol and is involved in androgen synthesis. When synthesis is active, few lipid droplets are observed and the volume is small. When synthesis is inactive, there are more lipid droplets and the volume is large. The number and volume of lipid droplets can be used to assess the function of Leydig cells. In the present, a large number of lipid droplets and a larger volume in Leydig cells were found in the DIO group compared with the control group. This change in lipid droplets in Leydig cells may reflect the decreased function of testosterone secreted by Leydig cells in the DIO group.

Increased apoptosis of Leydig cells was also found in this study. Apoptosis results from the activation of an intracellular program that leads to cell death without the induction of an inflammatory response. The increase in apoptosis of Leydig cells can reflect degenerative changes in Leydig cells, and the secretion of testosterone in Leydig cells is also decreased. There are numerous molecular pathways related to apoptosis. The primary effect of oxidative stress is on the mitochondrial membrane, where associations between pro- and antiapoptotic members of the Bcl-2 family (e.g., Bax and Bcl-X L or Bcl-2 and Bcl W, resp.) are altered [25, 26] allowing the release of cytochrome c and the eventual activation of a caspase cascade, which ultimately results in the fragmentation of cell DNA [27, 28]. Consistent with this pathway, Bax may be the predominant proapoptotic molecule in mouse testis where it may exhibit increased expression after obesity-induced oxidative stress. We will prove it in another experience. 

(*2) High Leptin Level and Low Testosterone Level Induced by Fat*. Leptin plays an important role in rodent and human reproduction [29, 30]. Recent research demonstrated that leptin directly inhibits human chorionic gonadotropin- (hCG-) stimulated testosterone secretion from rat Leydig cells in culture via a functional leptin receptor isoform and at concentrations within the range of obese men [31, 32]. Others have also shown that leptin inhibits basal and hCG-stimulated testosterone secretion in incubated rat testicular samples [33]. In addition, several studies have demonstrated that leptin levels are inversely correlated with testosterone [34–36]. This correlation is related to the suppressive effect of testosterone and its biologically active metabolite on leptin production [37]. In addition, it was recently proposed that testosterone may regulate ob gene expression [27]. The study by Isidori demonstrated that hyperleptinemia may have a role in the pathogenesis of reduced androgens in male obesity [38]. In this study, the serum concentration of leptin was inversely correlated with testosterone when mice were fed the high-fat diet for 8 or 19 weeks. Caprio et al. found that ob-R expression was present in embryonic, prepubertal, and adult rat testes. It is conceivable that high leptin concentration in males may have a direct inhibitory effect on Leydig cell function [32]. Leptin levels in obese human subjects are the best hormonal predictor of obesity-related reduction in androgen response to hCG tests in vivo [38]. The ob→ob-R system in the testis may negatively regulate testosterone production by Leydig cells [32]. Therefore, an excess of circulating leptin may be an important contributor to the development of reduced androgens in male obesity [38]. 

(*3) Oxidative Stress in Testicular Tissue and Low Level of Testosterone Induced by Fat Intake*. Many conditions or events associated with male infertility are inducers of oxidative stress and increasing testicular oxidative stress leads to an increase in germ cell apoptosis and subsequent hypospermatogenesis [18]. Several conditions related to male infertility whether therapeutic or pathological generate more reactive oxygen species (ROS) which are associated with reduced intracellular antioxidant activity unable to counter the ROS-mediated detrimental effect. Turner and Lysiak found that very large increases in NO were associated with oxidative stress, which may override the effects of hypoxia-inducible factor 1 (HIF-1) and inhibit testosterone production [18]. Testicular oxidative stress may be associated with decreased testosterone level. In this study, MDA, NO, and H_2_O_2_ increased and T-AOC (8w and 19w), CAT (8w and 19w), and GSH-Px (19w only) decreased in obese mice. The gene expression of these antioxidant enzymes was found to be reduced at 19 weeks (Figures 2(a)–2(d)). Increased NO levels are recognized as an indication of oxidative stress leading to inhibition of testosterone production [39]. There is evidence that H_2_O_2_ and NO besides acting as independent signaling molecules may interrelate to form an oxidative death cycle. H_2_O_2_ acts as an upstream signal leading to NO production [18, 40]. These results suggest that obesity-induced excessive oxidative stress production affected normal histological structures and function of testicular tissue.

Puberty is a complex process by which the androgen secreted by Leydig cells in testis tissue was response to hCG critically. And children develop secondary sexual characteristics and reproductive competence. The reproduction of a pubertal boy may be influenced by unhealthy environments. Taneli et al. demonstrated that obesity affects testicular Leydig cell function in obese adolescents according to pubertal stages [41]. In addition, increased oxidative stress in prepubertal severely obese children and obese adolescents has been reported [42, 43]. Vendramini et al. also found a reduction in sperm production in the pubertal phase in obese Zucker rats [12]. Increased oxidative stress and lipid peroxidation in testis tissue in pubertal obesity (due to fat accumulation) are extremely toxic to spermatozoa [44], Leydig cells in testis tissues may also be influenced, and androgen secretion may decrease. Secondary sexual characteristics and sexual maturity of males will be delayed by lower plasma testosterone. The reproduction of adult males will also be influenced. However, further investigation is needed to confirm these issues. These oxidants can cause tissue damage by a variety of mechanisms including DNA damage, lipid peroxidation, protein oxidation, depletion of cellular thiols, and activation of proinflammatory cytokine release [40]. The transcription factor, nuclear factor erythroid 2-related factor 2 (Nrf_2_), is a central regulator of antioxidant and detoxification gene expression in response to electrophilic or oxidative stress [45]. Nrf_2_, a member of the Cap “n” Collar family of basic region leucine zipper transcription factors, plays an important role in preventing the development of oxidative stress through upregulation of Nrf_2_-related antioxidants [46, 47]. Moreover, Nrf_2_-knockout mice showed an oxidative disruption in spermatogenesis [48]. In this study, the mRNA expression of Nrf_2_ decreased, as well as the activities and mRNA expression of enzymatic antioxidants. Thus, oxidative stress caused by high-fat diet in testes may be improved by reducing the Nrf_2_-antioxidant pathway.

It is interesting that reproductive hormone imbalance in obesity may affect the antioxidant status in testes. The immediate endocrine environment of the testes has a major impact on the antioxidant status of testes. Reproductive hormone imbalance, either hyper- or hypogonadotrophism, may contribute to the decline in antioxidant status in testes. Overstimulation of Leydig cells by chronic exposure to hCG (100 IU/day for 30 days in rats) also stimulates high levels of ROS production from these cells, which in turn stimulates lipid peroxidation, a reduction in antioxidant enzyme activities, germ cell apoptosis, and consequently disruption of spermatogenesis [49]. In contrast, research has shown that treatments including exposure to endocrine disruptors which diminish the intratesticular concentration of testosterone inhibit the testicular expression of antioxidant enzymes such as GPx, SOD, and catalase [50, 51]. When exogenous gonadotropin is administered to artificially elevate intratesticular testosterone levels, these suppressive effects on antioxidant expression, as well as the disruption of spermatogenesis, can be reversed [51, 52]. In this study, a low level of testosterone and high oxidative stress were observed in testis tissue. These findings further proved that hormone imbalance can aggravate oxidative stress in testis tissue in the obese state. However, further research on this topic is required.

In conclusion, short- (8 weeks) and long-term (19 weeks) high-fat diet increased Leydig cell pathological damage, apoptosis rates, lipid peroxidation, and serum leptin level; decreased sperm count, sperm motility, relative testis weight, and testosterone level; inhibited the activity of CAT and GSH-Px enzymes; decreased CAT, SOD, GSH-Px, and Nrf_2_ mRNA expression. Pubertal obesity may influence the function and development of reproductive organs in adults. Pathological damage of Leydig cells, oxidative stress in testis tissue, and high leptin level may provide some evidence to clarify the mechanisms of male SH in obesity and possibly prevent it.

## Figures and Tables

**Table 1:** Primer sequences for real-time RT-PCR analysis.

Gene	GenBank	Forward	Reverse
CAT	NM_009804	ACATGGTCTGGGACTTCTGG	CAAGTTTTTGATGCCCTGGT
GSH-Px	NM_008160	GTCCACCGTGTATGCCTTCT	TCTGCAGATCGTTCATCTCG
SOD	NM_0136713	TCAAGCGTGATTTGGGTCT	AGCGGAATAAGGCCTGTTGT
Nrf_2_	NM_172086	CTCGCTGGAAAAAGAAGTGG	CCGTCCAGGAGTTCAGAGAG

**Table 2:** The retroperitoneal, epididymal fat weight and reproductive organs weight in 8 weeks and 19 weeks ($\bar{x}$  ± SD).

	Group	*n*	Ret. fat (g)	Relative Ret. fat (g/100 g)	Epi. fat (g)	Relative Epi. fat (g/100 g)	Tes. weight (g)	Relative Tes. weight (g/100 g)	Epididymis weight (g)	Relative epididymis weight (g/100 g)	Sem. weight (g)	Relative Sem. weight (g/100 g)
8 weeks	DIO	12	0.09 ± 0.03**	0.34 ± 0.13**	0.32 ± 0.09**	1.23 ± 0.29**	0.19 ± 0.02	0.77 ± 0.06^∗#^	0.07 ± 0.01	0.29 ± 0.06	0.22 ± 0.02*	0.87 ± 0.11^##^
	DIO-R	12	0.08 ± 0.03**	0.37 ± 0.13**	0.32 ± 0.07**	1.49 ± 0.25**	0.18 ± 0.01	0.86 ± 00.07	0.07 ± 0.01	0.31 ± 0.02	0.24 ± 0.03**	1.12 ± 0.15
	Control	12	0.03 ± 0.01	0.12 ± 0.04	0.19 ± 0.04	0.91 ± 0.18	0.18 ± 0.03	0.85 ± 0.10	0.06 ± 0.01	0.31 ± 0.05	0.19 ± 0.03	0.91 ± 0.16^##^

19 weeks	DIO	12	0.49 ± 0.19**	1.49 ± 0.55**	1.15 ± 0.34**	3.52 ± 0.99**	0.20 ± 0.02	0.62 ± 0.08^∗∗##^	0.08 ± 0.01	0.25 ± 0.05**	0.31 ± 0.07	0.96 ± 0.21
	DIO-R	12	0.20 ± 0.06**	0.73 ± 0.22**	0.62 ± 0.19**	2.27 ± 0.67**	0.20 ± 0.01	0.72 ± 0.05	0.07 ± 0.01	0.27 ± 0.05	0.28 ± 0.060	1.02 ± 0.23
	Control	12	0.077 ± 0.037	0.28 ± 0.12	0.33 ± 0.06	1.19 ± 0.19	0.20 ± 0.02	0.75 ± 0.07	0.08 ± 0.01	0.30 ± 0.02	0.27 ± 0.06	1.01 ± 0.21

*Note.* Data are mean ± SD; *P < 0.05 and **P < 0.01 denote statistical significance compared with control group; ^#^ *P* < 0.05 and ^##^ *P* < 0.01 denote statistical significance compared with DIO-R group. Ret: retroperitoneal; Epi: epididymal; Tes: testis; Sem: seminal vesicles.

Relative ret. weight = retroperitoneal fat weight/body weight × 100. Relative Epi. fat weight = epididymal fat weight/body weight × 100. Relative Tes. weight = testis weight/body weight × 100. Relative epididymis weight = epididymis weight/body weight × 100. Relative Sem. weight = seminal vesicles weight/body weight × 100.

**Table 3:** Testosterone and leptin level in DIO, DIO-R, and control group ($\bar{x}$  ± SE).

Group	*n*	Testosterone (ng/mL)/8 W	Leptin (ng/mL)/8 W	Testosterone (ng/mL)/19 W	Leptin (ng/mL)/19 W
DIO	12	7.52 ± 0.25*	34.87 ± 4.37**	6.33 ± 0.56**	13.92 ± 1.96**
DIO-R	12	6.80 ± 0.23**	15.35 ± 2.69**	8.68 ± 1.68*	10.07 ± 1.37*
Control	12	10.81 ± 1.69	1.92 ± 0.34	12.68 ± 0.99	1.86 ± 0.24

*Note.* Data are mean ± SE. *P < 0.05 and **P < 0.01 denote statistical significance compared with control group.

**Table 4:** MDA, H_2_O_2_, T-AOC, GSH, SOD, GSH-Px, and CAT level of testis tissue in 8 weeks and 19 weeks ($\bar{x}$  ± SD).

	Group	*n*	MDA (nmol/mgprot)	T-AOC (U/mgprot)	SOD (U/mgprot)	GSH (mgGSH/gprot)	H_2_O_2_ (mmol/gprot)	CAT (U/mgprot)	GSH-Px (U/mgprot)	NO (umol/gprot)
8 weeks	DIO	12	0.38 ± 0.20**	1.03 ± 0.15	50.98 ± 10.26	12.78 ± 1.80	16.55 ± 3.73*	1.07 ± 0.31*	15.98 ± 3.10	0.32 ± 0.15**
	DIO-R	12	0.24 ± 0.12*	1.04 ± 0.20	49.71 ± 10.13	12.88 ± 1.66	16.75 ± 4.65*	0.98 ± 0.41*	13.71 ± 3.69	0.32 ± 0.10**
	Control	12	0.21 ± 0.07	0.94 ± 0.13	53.45 ± 14.64	13.09 ± 1.98	10.82 ± 1.16	1.52 ± 0.25	15.93 ± 3.36	0.19 ± 0.03

19 weeks	DIO	12	0.89 ± 0.24*	0.73 ± 0.23*	71.46 ± 10.63	13.38 ± 1.20*	16.68 ± 4.35*	0.28 ± 0.08*	26.26 ± 6.34*	0.32 ± 0.13**
	DIO-R	12	1.12 ± 0.51**	0.77 ± 0.24	76.97 ± 16.74	12.84 ± 2.81	15.58 ± 2.98	0.15 ± 0.07**	26.03 ± 6.34*	0.27 ± 0.06*
	Control	12	0.47 ± 0.12	0.94 ± 0.16	71.94 ± 7.56	10.92 ± 2.48	12.62 ± 2.69	0.53 ± 0.24	33.52 ± 5.42	0.18 ± 0.05

*Note.* Data are mean ± SD. *P < 0.05 and **P < 0.01 denote statistical significance compared with control group.

## References

[1] Mendez R, Grissom M (2013). Disorders of childhood growth and development: childhood obesity. *FP Essentials*.

[2] Ma J, Cai CH, Wang HJ, Dong B, Song Y, Hu PJ (2012). The trend analysis of overweight and obesity in Chinese students during 1985 to 2010. *Chinese Journal of Preventive Medicine*.

[3] Han JC, Lawlor DA, Kimm SY (2010). Childhood obesity. *The Lancet*.

[4] Flegal KM, Wei R, Ogden CL, Freedman DS, Johnson CL, Curtin LR (2009). Characterizing extreme values of body mass index-for-age by using the 2000 Centers for Disease Control and Prevention growth charts. * The American Journal of Clinical Nutrition*.

[5] Freedman DS, Mei Z, Srinivasan SR, Berenson GS, Dietz WH (2007). Cardiovascular risk factors and excess adiposity among overweight children and adolescents: the Bogalusa Heart study. *Journal of Pediatrics*.

[6] Koebnick C, Smith N, Coleman KJ (2010). Prevalence of extreme obesity in a multiethnic cohort of children and adolescents. *Journal of Pediatrics*.

[7] Johnston CA, Tyler C, Palcic JL, Stansberry SA, Gallagher MR, Foreyt JP (2011). Smaller weight changes in standardized body mass index in response to treatment as weight classification increases. *Journal of Pediatrics*.

[8] Savoye M, Nowicka P, Shaw M (2011). Long-term results of an obesity program in an ethnically diverse pediatric population. *Pediatrics*.

[9] Danielsson P, Kowalski J, Ekblom Ö, Marcus C (2012). Response of severely obese children and adolescents to behavioral treatment. *Archives of Pediatrics and Adolescent Medicine*.

[10] Dandona P, Dhindsa S (2011). Update: hypogonadotropic hypogonadism in type 2 diabetes and obesity. *Journal of Clinical Endocrinology and Metabolism*.

[11] Hammoud AO, Wilde N, Gibson M, Parks A, Carrell DT, Meikle AW (2008). Male obesity and alteration in sperm parameters. *Fertility and Sterility*.

[12] Vendramini V, Cedenho AP, Miraglia SM, Spaine DM (2014). Reproductive function of the male obese Zucker rats: alteration in sperm production and sperm DNA damage. *Reproductive Science*.

[13] Mammi C, Calanchini M, Antelmi A (2012). Androgens and adipose tissue in males: a complex and reciprocal interplay. *International Journal of Endocrinology*.

[14] Hofstra J, Loves S, van Wageningen B, Ruinemans-Koerts J, Janssen I, de Boer H (2008). High prevalence of hypogonadotropic hypogonadism in men referred for obesity treatment. *Netherlands Journal of Medicine*.

[15] Saboor Aftab SA, Kumar S, Barber TM (2013). The role of obesity and type 2 diabetes mellitus in the development of male obesity-associated secondary hypogonadism. *Clinical Endocrinology*.

[16] Esposito K, Giugliano D (2011). Obesity, the metabolic syndrome, and sexual dysfunction in men. *Clinical Pharmacology and Therapeutics*.

[17] Gerber GS, Brendler CB, Wein AJ (2007). Evaluation of the urologic patient: history, physical examination, and the urinalysis. *Campbell-Walsh Urology*.

[18] Turner TT, Lysiak JJ (2008). Oxidative stress: a common factor in testicular dysfunction. *Journal of Andrology*.

[19] Gu H, Liu L, Ma S (2009). Inhibition of SOCS-3 in adipocytes of rats with diet-induced obesity increases leptin-mediated fatty acid oxidation. *Endocrinology*.

[20] Levin BE, Keesey RE (1998). Defense of differfing body weight set points in diet-induced obese and resistant rats. *The American Journal of Physiology: Regulatory Integrative and Comparative Physiology*.

[21] Deng Y, Xu ZF, Liu W, Xu B, Yang HB, Wei YG (2012). Riluzole-triggered GSH synthesis via activation of glutamate transporters to antagonize methylmercury-induced oxidative stress in rat cerebral cortex. *Oxidative Medicine and Cellular Longevity*.

[22] WHO (1999). *WHO Laboratory Manual for the Examination of Human Semen and Sperm-Cervical Mucus Interaction*.

[23] Alshabanah OA, Hafez MM, Al-Harbi MM (2010). Doxorubicin toxicity can be ameliorated during antioxidant L-carnitine supplementation. *Oxidative Medicine and Cellular Longevity*.

[24] de Maddalena C, Vodo S, Petroni A, Aloisi AM (2012). Impact of testosterone on body fat composition. *Journal of Cellular Physiology*.

[25] Adams JM, Cory S (1998). The Bcl-2 protein family: arbiters of cell survival. *Science*.

[26] Hengartner MO (2000). The biochemistry of apoptosis. *Nature*.

[27] Green DR (1998). Apoptic pathways: the roads to ruin. *Cell*.

[28] Wyllie AH (1980). Glucocorticoid-induced thymocyte apoptosis is associated with endogenous endonuclease activation. *Nature*.

[29] Cravo RM, Frazao R, Perello M (2013). Leptin signaling in Kiss1 neurons arises after pubertal development. *PLoS ONE*.

[30] Zuure WA, Roberts AL, Quennell JH, Anderson GM (2013). Leptin signaling in GABA neurons, but not glutamate neurons, is required for reproductive function. *The Journal of Neuroscience*.

[31] Caprio M, Isidori AM, Carta AR, Moretti C, Dufau ML, Fabbri A (1999). Expression of functional leptin receptors in rodent Leydig cells. *Endocrinology*.

[32] Caprio M, Fabbrini E, Ricci G (2003). Ontogenesis of leptin receptor in rat Leydig cells. *Biology of Reproduction*.

[33] Tena-Sempere M, Pinilla L, González LC, Diéguez C, Casanueva FF, Aguilar E (1999). Leptin inhibits testosterone secretion from adult rat testis in vitro. *Journal of Endocrinology*.

[34] Vettor R, De Pergola G, Pagano C (1997). Gender differences in serum leptin in obese people: relationships with testosterone, body fat distribution and insulin sensitivity. *European Journal of Clinical Investigation*.

[35] Luukkaa V, Pesonen U, Huhtaniemi I (1998). Inverse correlation between serum testosterone and leptin in men. *Journal of Clinical Endocrinology and Metabolism*.

[36] Wabitsch M, Blum WF, Muche R (1997). Contribution of androgens to the gender difference in leptin production in obese children and adolescents. *Journal of Clinical Investigation*.

[37] Xi H, Zhang L, Guo Z, Zhao L (2011). Serum leptin concentration and its effect on puberty in Naqu Tibetan adolescents. *Journal of Physiological Anthropology*.

[38] Isidori AM, Caprio M, Strollo F (1999). Leptin and androgens in male obesity: evidence for leptin contribution to reduced androgen levels. *Journal of Clinical Endocrinology and Metabolism*.

[39] Mehta A, Sekhon CPS, Giri S, Orak JK, Singh AK (2002). Attenuation of ischemia/reperfusion induced MAP kinases by N-acetyl cysteine, sodium nitroprusside and phosphoramidon. *Molecular and Cellular Biochemistry*.

[40] Anand H, Misro MM, Sharma SB, Prakash S (2013). Cytoprotective effects of fruit pulp of Eugenia jambolana on H_2_O_2_-induced oxidative stress and apoptosis in rat Leydig cells in vitro. *Andrologia*.

[41] Taneli F, Ersoy B, Özhan B (2010). The effect of obesity on testicular function by insulin-like factor 3, inhibin B, and leptin concentrations in obese adolescents according to pubertal stages. *Clinical Biochemistry*.

[42] Montero D, Walther G, Perez-Martin A, Roche E, Vinet A (2012). Endothelial dysfunction, inflammation, and oxidative stress in obese children and adolescents: markers and effect of lifestyle intervention. *Obesity Reviews*.

[43] Mohn A, Catino M, Capanna R, Giannini C, Marcovecchio M, Chiarelli F (2005). Increased oxidative stress in prepubertal severely obese children: effect of a dietary restriction-weight loss program. *Journal of Clinical Endocrinology and Metabolism*.

[44] Teerds KJ, de Rooij DG, Keijer J (2011). Functional relationship between obesity and male reproduction: from humans to animal models. *Human Reproduction Update*.

[45] Chartoumpekis DV, Ziros PG, Zaravinos A (2013). Hepatic gene expression profiling in Nrf2 knockout mice after long-term high-fat diet-induced obesity. *Oxidative Medicine and Cellular Longevit*.

[46] Cui W, Li B, Bai Y (2013). Potential role for Nrf2 activation in the therapeutic effect of MG132on diabetic nephropathy in OVE26 diabetic mice. *The American Journal of Physiology: Endocrinology and Metabolism*.

[47] Sporn MB, Liby KT (2012). NRF2 and cancer: the good, the bad and the importance of context. *Nature Reviews*.

[48] Nakamura BN, Lawson G, Chan JY (2010). Knockout of the transcription factor NRF2 disrupts spermatogenesis in an age-dependent manner. *Free Radical Biology and Medicine*.

[49] Gautam DK, Misro MM, Chaki SP, Chandra M, Sehgal N (2007). hCG treatment raises H_2_O_2_ levels and induces germ cell apoptosis in rat testis. *Apoptosis*.

[50] Zini A, Schlegel PN (2003). Effect of hormonal manipulation on mRNA expression of antioxidant enzymes in the rat testis. *Journal of Urology*.

[51] Ghosh D, Das UB, Ghosh S, Mallick M, Debnath J (2002). Testicular gametogenic and steroidogenic activities in cyclophosphamide treated rat: a correlative study with testicular oxidative stress. *Drug and Chemical Toxicology*.

[52] Peltola V, Huhtaniemi I, Metsa-Ketela T, Ahotupa M (1996). Induction of lipid peroxidation during steroidogenesis in the rat testis. *Endocrinology*.

